# Light Scattering By Optically-Trapped Vesicles Affords Unprecedented Temporal Resolution Of Lipid-Raft Dynamics

**DOI:** 10.1038/s41598-017-08980-1

**Published:** 2017-08-17

**Authors:** Liam Collard, David Perez-Guaita, Bayan H. A. Faraj, Bayden R. Wood, Russell Wallis, Peter W. Andrew, Andrew J. Hudson

**Affiliations:** 1 Department of Chemistry, University of Leicester, Leicester, LE1 7RH United Kingdom; 2 Department of Mathematics, University of Leicester, Leicester, LE1 7RH United Kingdom; 3 Department of Chemistry, Monash University, Clayton, Victoria, 3800 Australia; 4 Department of Infection, Immunity and Inflammation, University of Leicester, Leicester, LE1 9HN United Kingdom; 5 Department of Molecular and Cell Biology, University of Leicester, Leicester, LE1 7RH United Kingdom

## Abstract

A spectroscopic technique is presented that is able to identify rapid changes in the bending modulus and fluidity of vesicle lipid bilayers on the micrometer scale, and distinguish between the presence and absence of heterogeneities in lipid-packing order. Individual unilamellar vesicles have been isolated using laser tweezers and, by measuring the intensity modulation of elastic back-scattered light, changes in the biophysical properties of lipid bilayers were revealed. Our approach offers unprecedented temporal resolution and, uniquely, physical transformations of lipid bilayers can be monitored on a length scale of micrometers. As an example, the deformation of a membrane bilayer following the gel-to-fluid phase transition in a pure phospholipid vesicle was observed to take place across an interval of 54 ± 5 ms corresponding to an estimated full-width of only ~1 m°C. Dynamic heterogeneities in packing order were detected in mixed-lipid bilayers. Using a ternary mixture of lipids, the modulated-intensity profile of elastic back-scattered light from an optically-trapped vesicle revealed an abrupt change in the bending modulus of the bilayer which could be associated with the dissolution of ordered microdomains (*i.e*., lipid rafts). This occurred across an interval of 30 ± 5 ms (equivalent to ~1 m°C).

## Introduction

The fluid-mosaic model describes the self-organisation, and lateral and rotational diffusion, of lipids and proteins embedded in the bilayer structure of cellular membranes^1^. Refinement of the model has been made to account for non-homogeneous diffusion leading to the segregation of lipid components^2^, and it has been postulated that ordered microdomains (lipid rafts) could be involved in membrane signalling and trafficking^3^. Although ordered microdomain structures, enriched in cholesterol and sphingolipids, have been observed to float freely in the fluid matrix of artificial membranes, there is still controversy surrounding the dimensions of lipid rafts, and whether or not their existence is only transient in cellular membranes. A challenge to studying membrane processes is sensitivity to structural, or biochemical, changes that might be localised in ordered microdomains. Due to the lack of suitable techniques, the majority of studies of membrane processes have concentrated on protein-protein interactions in bilayers, and either changes in bilayer structure induced by membrane proteins, or the influence of microdomain structure on protein interactions, have largely been neglected. One example is pore formation by cholesterol-dependent cytolysins. The protein is assumed to be sequestered to microdomains enriched in cholesterol^4^; the mechanism of protein-protein interactions resulting in self-assembly of large transmembrane β-barrel oligomers has been characterised^4–7^, but the role of target membranes in the self assembly mechanism has received comparatively little attention. In the work described here, we demonstrate that existence of microdomain structure in a single lipid vesicle of 1 μm diameter can be deduced. The potential to observe the heterogeneities in lipid ordering in a liposomal membrane could help to provide information on how lipid-lipid and lipid-protein interactions change during the self assembly of protein oligomers.

Raman-scattered light can be efficiently collected from a lipid vesicle isolated using optical tweezers^8, 9^, and the positions and relative intensities of peaks in the C-H stretching region of Raman spectra can be reconciled with membrane-lipid order^10, 11^. Harris and co-workers have measured the Raman spectrum from a single, optically-trapped, vesicle of a saturated lipid as a function of temperature; and, by using multivariate analysis, were able to deconvolve the spectral data into components for the sub-gel, gel, ripple and fluid phases^12^. The same group have reported a wide range of data on the biophysical properties of lipid membranes using similar methodology^13–16^. De Wit *et al*. have shown that light scattering intensities are dependent on lipid phases in droplet-interface bilayers, and were able to image the dynamic nature of lipid rafts on the millisecond timescale^17^.

This paper reports on the determination of changes in the bending modulus and fluidity of lipid bilayers in isolated unilamellar vesicles by measuring the modulated intensity of elastic back-scattered light. Due to the dependence of light scattering intensity on bilayer fluidity, the technique has the sensitivity to reveal dynamic heterogeneities in lipid-packing order in a liposomal bilayer (surface area of ~3 μm^2^). Morphological changes to the lipid vesicle that are a consequence of the formation or dissolution of microdomains in a fluid bilayer, or transitions between structures resembling the ideal lamellar phases, have been observed with unprecedented temporal resolution. The new methodology complements Raman measurements which have been widely used to report on the short-range packing order of the hydrocarbon chains and rotational diffusion of lipids in bilayers. The spectral distribution of Raman-scattered light provides information on the environment of the lipid molecules, and the elastic-light scattering intensity reports on the bending modulus and fluidity in multicomponent vesicle lipid bilayers. While the elastic back-scattered light was modulated sharply in mixed-lipid bilayers, the frequencies and relative intensities of the C-H stretching bands, exhibited a more gradual change as temperature was increased.

## Results and Discussion

Unilamellar lipid vesicles were prepared by extrusion and the experiments were performed using a customised-inverted microscope with a heated stage, and capabilities for optical tweezing (λ_trap_, 1070 nm), Raman microspectroscopy (λ_excitation_, 488 nm) and intensity measurement of elastic back-scattered light. The time-dependent modulation of the intensity of back-scattered light from a trapped liposome was monitored by recording either the IR wavelength (1070 nm) used to create the optical tweezers, or the visible wavelength from the Raman excitation laser (488 nm). The scattering signal at 1070 nm could be monitored at 0.25 s intervals by an ordinary photodiode (0.1 ms rise-time response), and the signal at 488 nm could be monitored at 4 μs intervals by an avalanche photodiode (20 ns rise-time response). The optical trapping laser was maintained at the lowest power level capable of holding the liposome for the duration of an experiment that involved a temperature ramp between 20 and 60 °C.

**Figure 1:**
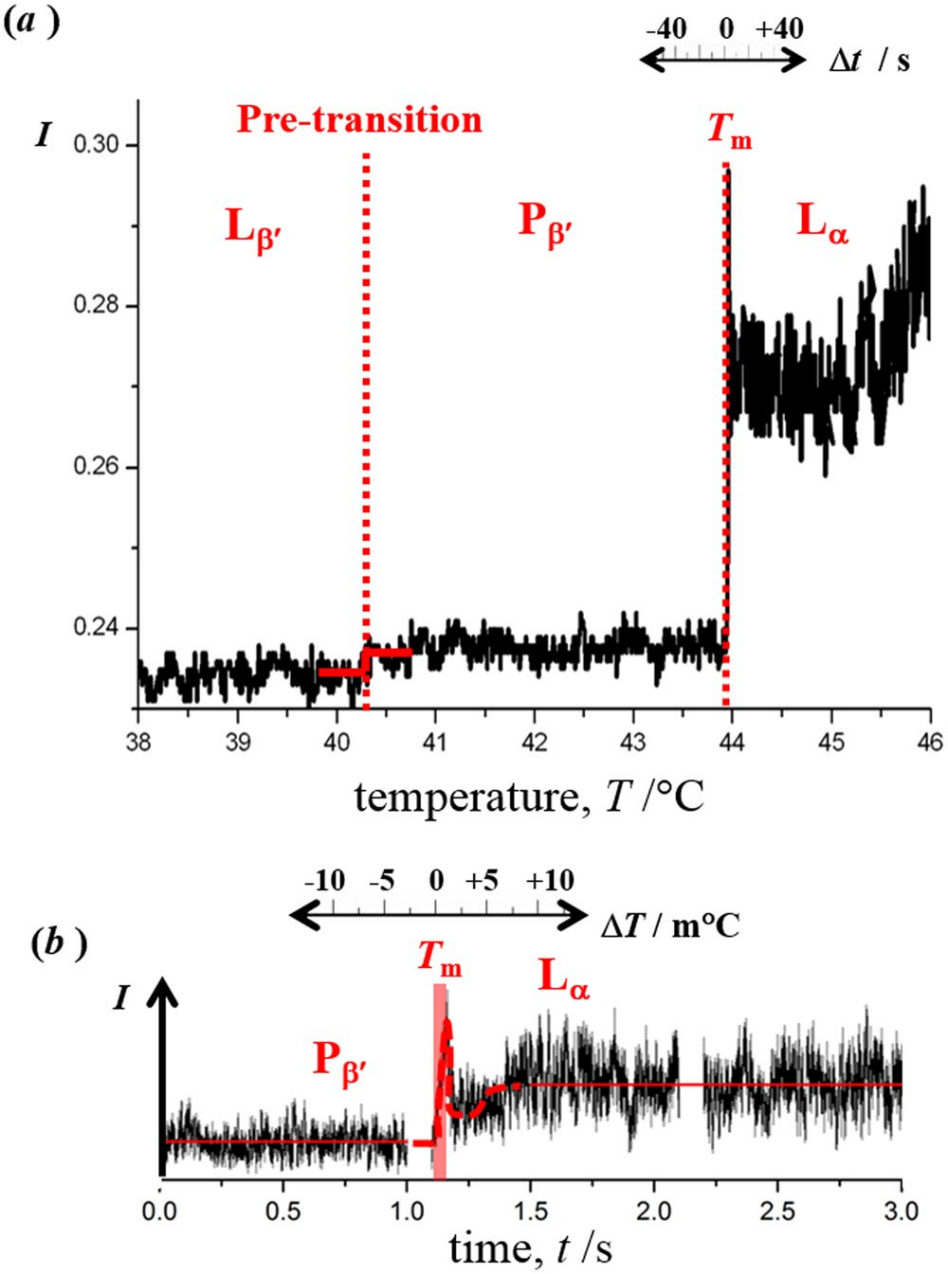
An optically-trapped DPPC liposome. (**a**) The back-scattered intensity at 1070 nm. (**b**) The back-scattered intensity of light at 488 nm measured, with high temporal resolution, across the main transition, L_β′_ → L_α_. The horizontal red line shown in (**b**) represents the average intensity signal across the time range.

Figure 1(a) shows the temperature dependence of the intensity of back-scattered light, at 1070 nm, from an optically-trapped liposome of pure 1,2-dipalmitoyl**-**sn**-**glycero-3-phosphocholine (DPPC, 1 μm-diameter). A temperature ramp, from 20 to 60 °C, was applied that incorporates the regions of the pre-transition (gel to ripple phase, L_β′_→P_β′_) and the main transition of the lipid bilayer (ripple to fluid phase, P_β′_→L_α_). We have not explored a temperature range incorporating the sub-transition, occurring at ~13.5 °C, from the L_c_ phase with crystalline-like packing of DPPC molecules to the gel phase, L_β′_^18^. In the vicinity of the main transition, the ramp was approximately +29 m°C s^−1^. The heating rate was not regulated, leading to a non-linear increase but minimising transient fluctuations in temperature. The profile of the temperature ramp for the experiment illustrated in 1(a) is provided in the Supporting Information (SI; see Figure S2). The pre-transition is accompanied by a small change in the intensity of back-scattered light (full width, 0.14 °C). The main transition is accompanied by a sharp change in intensity, which was detected in successive measurements of the back-scattered intensity separated by 0.25 s (see 1(*a*)). Tentatively it could be claimed that the full width was only ~7 m°C for the single liposome, however, it is conceivable that small transient fluctuations in temperature were superimposed on the approximately-linear ramp (see S2). Still, the temporal resolution of the optical tweezing apparatus significantly exceeds the capabilities of conventional methods to detect physical changes in lipid bilayers that accompany the phase transitions, and provides the unique opportunity to probe the physical properties of a single vesicle. The example shown in 1 (*a*) is representative of the light scattering traces recorded for 7 different liposomes; see Figures S5 and S7 in the SI. A sharp change in the light-scattering intensity accompanying the reverse (fluid-to-gel) phase transition, L_α_ → P_β′_, L_β′_, could not be observed (see examples in S7). The only explanation for the absence of an abrupt change is supercooling of the L_α_ phase of the vesicle. This effect leads to a dramatic difference between the heating and cooling profiles for light scattering in comparison to the minor hysteresis in the main transition temperature observed by differential scanning calorimetry. The reason that the difference is pronounced must be the result of the small amount of lipid present in a single isolated vesicle, the relative high rate of cooling in the light scattering experiment and the absence of large numbers of discontinuities or defects in the lipid bilayer structure of a single vesicle. The light-scattering measurement would be insensitive to a liquid-to-gel transition that is delayed (by minutes) as the result of supercooling.

Light scattering has previously been shown to have the capability to monitor the main transition in a bulk suspension of DPPC liposomes, where the intensity at a fixed angle was observed to change across a broad interval of 1 °C^19^. A more precise determination of the transition can be made by differential dilatometry; a half width of only 0.15 °C was reported in ref. 20. The heating rate used in the light-scattering experiment reported in **1(a)** corresponds to 1.7 °C min^−1^ (*i.e*. 0.029 °C s^−1^) which is high in comparison to typical rates used in standard thermal analysis. In the aforementioned study by differential dilatometry, the authors employed a temperature ramp of 1 °C hr^−1^ and observed an abrupt change in the thermal expansion of lipid suspensions from approximately 8 × 10^6^ ml g^−1^ °C^−1^ to 1.2 × 10^9^ ml g^−1^ °C^−1^, over a 9 minute interval (corresponding to 0.15 °C). Alaouie *et al*. have also recorded the thermograms by differential scanning calorimetry (DSC) for 1,2-ditetradecanoyl-*sn*-glycero-3-phosphocholine (DMPC) bilayers (which has a main transition temperature of ~23 °C)^21^. The authors measured the profile of the heat flow as a function of the ramp rate from 0.1 °C min^−1^ to 1.75 °C min^−1^; at the lowest rate, a full-width-half-maximum (FWHM) height of 0.29 °C was observed over an interval of 171 s; and, at the highest rate, a FWHM of 1.7 °C was observed over an interval of 59 s. The highest rate of measurement by DSC corresponds to the conditions in the experiments reported here; however, in Alaouie *et al*.^21^, a 20 μL sample was used but the profile in 1(a) was obtained from a 1 fL volume (encompassing a single liposomal bilayer), and the temporal and temperature width of the transition is remarkably narrower.

**Figure 2:**
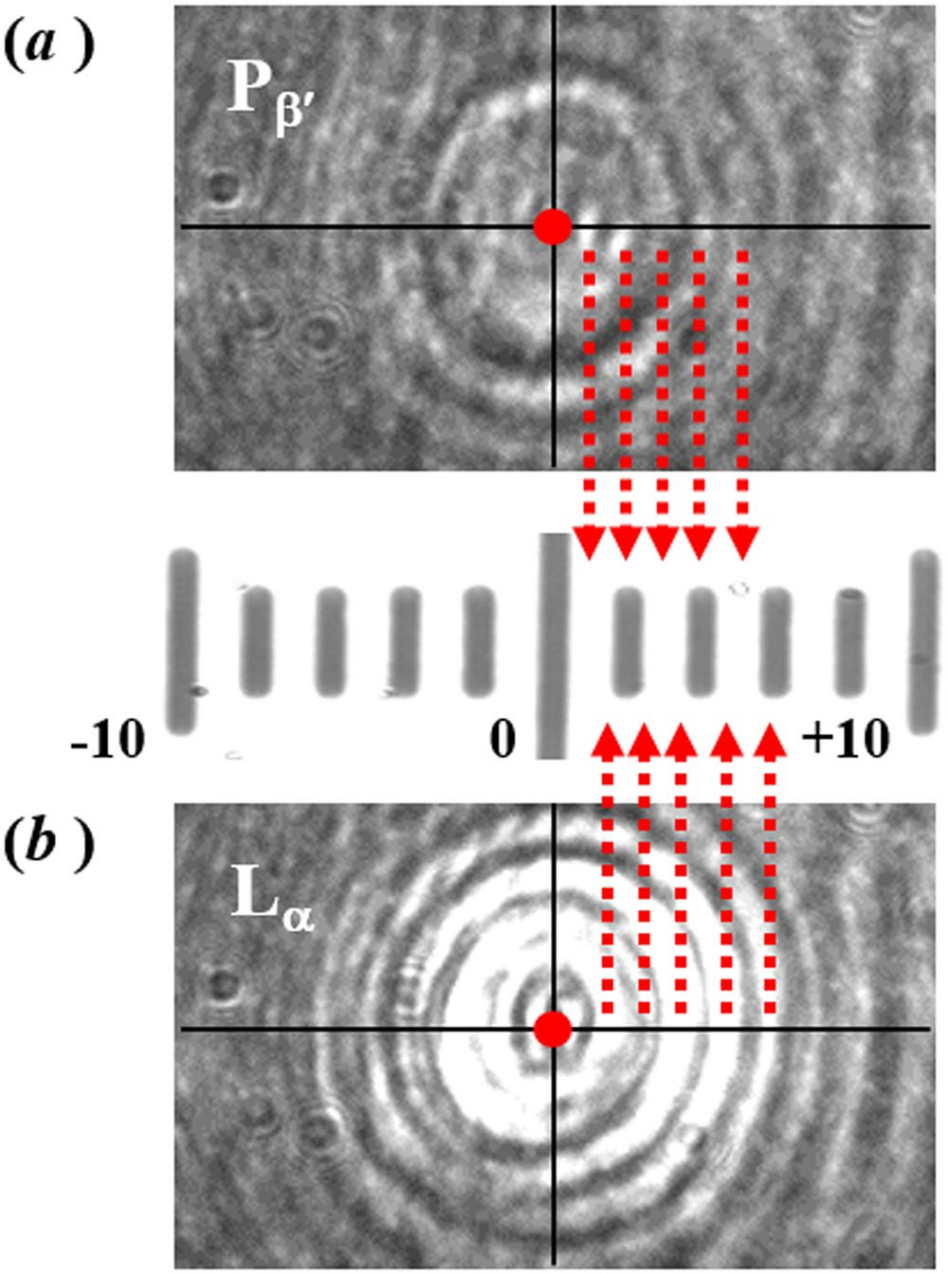
Images of the back-scattered light, at 1070 nm, from an optically-trapped DPPC liposome. Snapshots of the diffraction pattern of scattered light were recorded at 1 s interval during a temperature ramp. The images shown were recorded (**a**) immediately before, and (**b**) immediately after, a dramatic change in the overall scattering intensity was observed; i.e. across the P_β′_ → L_α_ transition. A bright field image of a graticule (−10 to +10 μm) is reproduced in the centre panel.

A further determination of the main transition was made at higher temporal resolution by recording the intensity of back-scattered light from a 488 nm laser on a fast avalanche photodiode. In this measurement, the abrupt change in the signal intensity was observed over 54 ± 5 ms (*i.e*. the minimum-to-maximum intensity in 1(*b*)). If it is assumed that there are no transient fluctuations in temperature in the region of the optically-trapped liposome, this timescale corresponds to a full width of ~1 m°C, which is much lower than the values observed for other physical parameters such as the heat flow and volume. Both light-scattering profiles, in 1(*a*) and (*b*), show an increase in intensity following the main transition, P_β′_ → L_α_. An increase in the back-scattered intensity appears to be a consequence of deformation of the lipid membrane during the course of the phase transition, resulting in a larger area of lipid bilayer located in regions of higher power density of the trapping laser; and a smaller vesicle diameter along the equatorial axis. Images of the light scattering profile, recorded on a charge-coupled device, from an optically-trapped DPPC liposome are shown in Fig. 2 just below and just above the temperature where the dramatic change in the intensity of light scattering is detected. The acquisition rate was 1 frame per second with an 80 ms integration time. The change in the diameter of the diffraction rings observed in the images support the conclusion that the light scattering change could arise from deformation of the lipid membrane with an increase in the polar length and a concomitant decrease in the equatorial length. In the intensity-time profile shown in 1(*a*), there is an increase in signal-to-noise immediately following the phase transition. This is likely to be due to shape fluctuations of the DPPC-liposome occurring after the bilayer has transitioned into the fluid phase.

The spectrum of inelastic (Raman) scattered-light from an optically-trapped DPPC liposome measured in the gel (L_β′_), ripple (P_β′_) and fluid (L_α_) phases has been reported by others^10–12, 22^. The full width of the gel-to-fluid transition, which is illustrated in Fig. 1 is significantly narrower than the temperature modulation of Raman spectra reported in the literature^12, 22^. The broad temperature range over which the gel-to-fluid transition is observed in these reports is not a technical limitation of the Raman technique, but it is inherent in how the Raman spectra report on changes in bilayer structure around the main transition. Spectra similar to those earlier measurements have been recorded and are shown in the SI. The C-H region comprises the symmetric (d^+^) and antisymmetric (d^−^) methylene stretch, the Fermi resonance of the symmetric methyl stretch (r_FR_^+^) and the antisymmetric methyl stretch (r^−^)^23^. The intensity ratio between the d^−^ and d^+^ bands, or, alternatively, the d^−^ and r_FR_^+^ bands, has been interpreted in the literature as a qualitative measure of short-range packing order of the hydrocarbon chains, where a larger ratio is indicative of greater order^24^. As expected, a decrease in both of these ratios is observed above the main transition for a single DPPC liposome.

Binary mixtures of a phosphocholine lipid and cholesterol (chol) have been proposed to exhibit a liquid-ordered phase (L_o_) sharing characteristics of gel (L_β′_) and fluid (liquid-disordered, L_d_) phases. While the intercalation of chol with phosphocholine molecules disrupts the planar triangular lattice in the gel phase, a *trans* conformation of the hydrocarbon chains is more favoured in the fluid phase resulting in a higher degree of short-range order. A 1:1 mole ratio of lipid components, at 20 °C, produces a bilayer in the L_o_ phase; the L_o_ and L_d_ phases are expected to co-exist in bilayers at either lower mole fractions of chol or, alternatively, at higher temperature^25^. The temperature-dependent phase behaviour of POPC/chol bilayers was inferred in ref. 25 from measurements of membrane fluidity; however, an enthalpy change cannot be detected between 20 and 60 °C by differential scanning calorimetry (see SI) and, hence, a thermotropic phase transition, L_o_ → L_o_/L_d_, cannot be confirmed. Co-existing L_o_ and L_d_ phases are expected to exist at room temperature for a ternary mixture of a phosphocholine lipid (1-palmitoyl-2-oleoyl-sn-glycero-3-phosphocholine, POPC), chol and sphingomyelin (SM) in a 1:1:1 mole ratio. This composition is widely used as a system for observation of lipid rafts, which are microdomains proposed to resemble the structure of the L_o_ phase enriched in both chol and SM. The growth and dissolution of lipid rafts in the fluid-L_d_ matrix is highly dynamic^26^. Despite the resemblance between structures of lipid rafts and the L_o_ phase, lipid rafts are not the result of thermotropic phase separation from the L_d_ bilayer. A change from co-existing L_o_/L_d_ phases to a homogeneous L_d_ phase has been predicted to take place at higher temperature based on measurements of membrane fluidity^25^; however, an enthalpy change is not revealed by differential scanning calorimetry (see SI). There is not sufficient evidence in the literature to suggest that the occurrence of lipid rafts should be limited to thermodynamic conditions of L_o_/L_d_ phase co-existence^26^.

**Figure 3:**
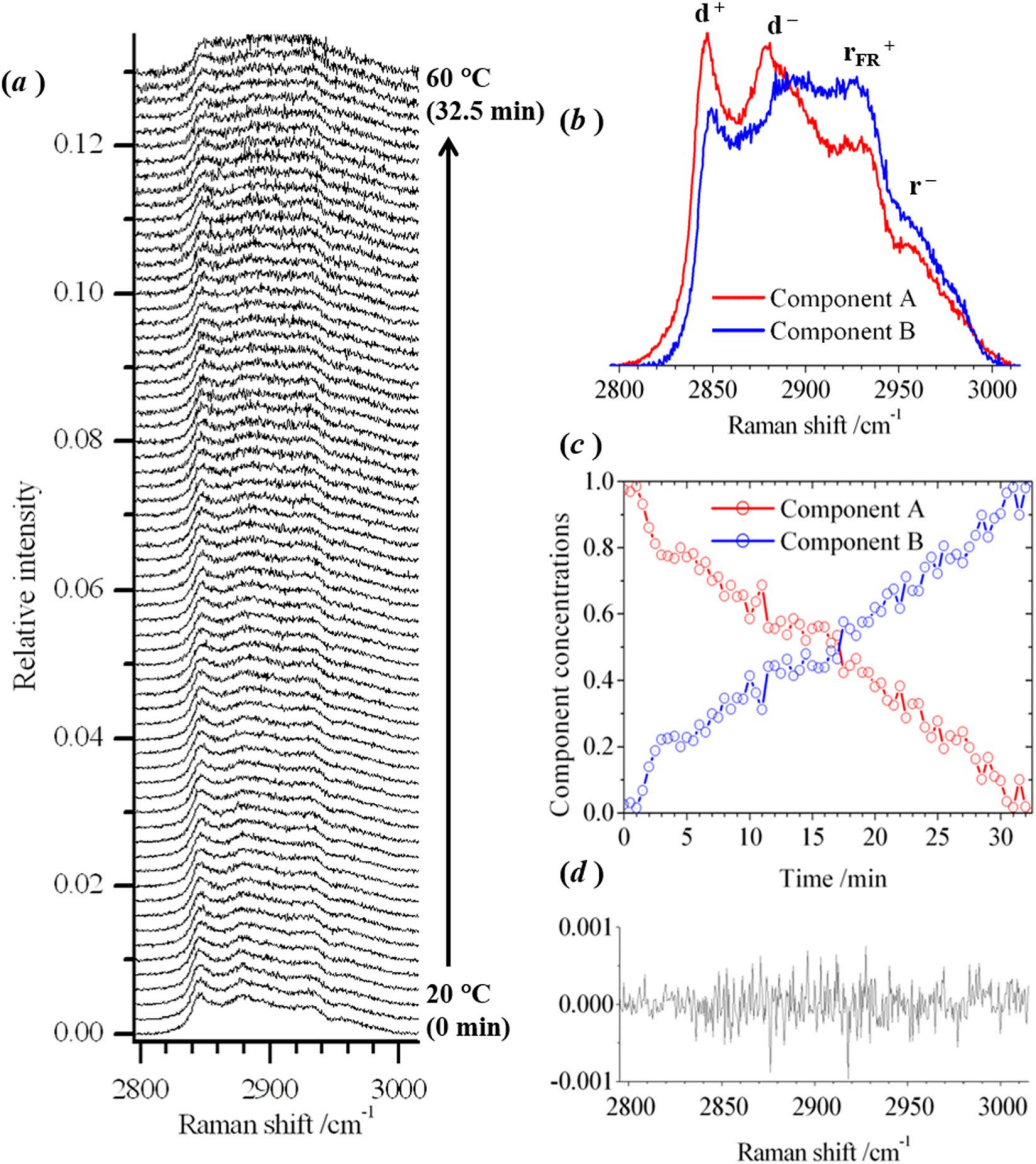
Raman spectra of an optically-trapped POPC/chol/SM liposome, in the region of the C-H stretching band. (**a**) A sequence of experimental Raman spectra recorded at 30 s intervals during a temperature ramp between 20 and 60 °C. (**b**) and (**c**) The pure spectral profiles and concentration profiles for two components obtained by multivariate curve resolution. **(d)** The residual for the 35^th^ spectrum, recorded after 17 minutes.

1:1 and 1:1:1 compositions for binary POPC/chol and ternary POPC/chol/SM bilayers, respectively, have been studied using elastic and inelastic light-scattering measurements from optically-trapped liposomes. In Fig. 3(a), the Raman spectrum of an optically-trapped POPC/chol/SM liposome is shown as a function of temperature. All the spectra were recorded from the same trapped vesicle at regular intervals of time during a temperature ramp from 20 to 60 °C. Data for the binary mixture of POPC/chol is shown in the SI; further detail on lipid-structure related interpretation of Raman spectra is also provided in the SI. The d^+^, d^−^, r_FR_^+^ and r^−^ bands can be distinguished in the broad C-H region, but the change in the structure of the lipid bilayer between room temperature, where L_o_ and L_d_ phases are predicted to co-exist, and high temperature, where a structure resembling the pure L_d_ phases is predicted, appears to result in a small increase in the ratio d^−^/d^+^. This is not the direction of change that was expected for the ratio of the CH_2_ stretching bands. A decrease in the ratio is usually observed with a loss in short-range order of the hydrocarbon chain packing^10–12^. Although there is considerable analysis and interpretation of the temperature-dependence of Raman spectra in the vicinity of the main transition in lipid bilayers, there is relatively sparse reported data on the structural changes in fluid multicomponent systems. The apparent increase d^−^/d^+^ in Fig. 3 indicates that it is not strictly reliable to associate the relative intensities of Raman peaks with lipid ordering in bilayer membranes. Although the change in the ratio d^−^/d^+^ with temperature is not typical, a shift in the d^-^ band to higher frequency can be seen in the raw spectral profiles in 3(*a*) which is consistent with interpretation in the literature. The d^−^ frequency has previously been associated with the extent of chain decoupling and rotational diffusion of lipids^10^. Furthermore, there is a substantial increase in the intensity of the r_FR_^+^ band relative to the d^-^ band in 3(*a*), which has also been used as an indicator of packing disorder^10^.

An alternative to using Raman-peak intensities to make a direct interpretation of the physical properties of lipid bilayers is to apply chemometric methods to decompose each spectrum into underlying components: a procedure that was used, in ref. 12, to derive spectral profiles and temperature-dependent concentrations for component sub-gel, gel, ripple and fluid phases of DPPC bilayers. The results of multivariate curve resolution alternate-least squares analysis on the sequence of Raman spectra from Fig. 3(a) is shown in 3(*b*) and (*c*). A fitting of two components captured 99.4% of the variance in the experimental data. In 3(*d*), the residual is shown for a representative example from the sequence of experimental spectra in 3(*a*). The signal remaining in 3(*d*) appears to be stochastic, without any trace of the C-H stretching band, and hence supports the suitability of the multivariate model. The chemometric analysis suggests that the Raman intensity profile changes continuously, and smoothly, between 20 and 60 °C. The fitted components, A and B, bear a close resemblance to the initial and final experimental spectra, respectively. A similar result was obtained for 1:1 POPC/chol bilayers (see SI). A control experiment was also performed, in which a sequence of Raman spectra were recorded from an optically-trapped vesicle maintained at ambient temperature (SI). No change in the frequencies or intensities of peaks in the C-H stretching region was observed at constant temperature.

**Figure 4:**
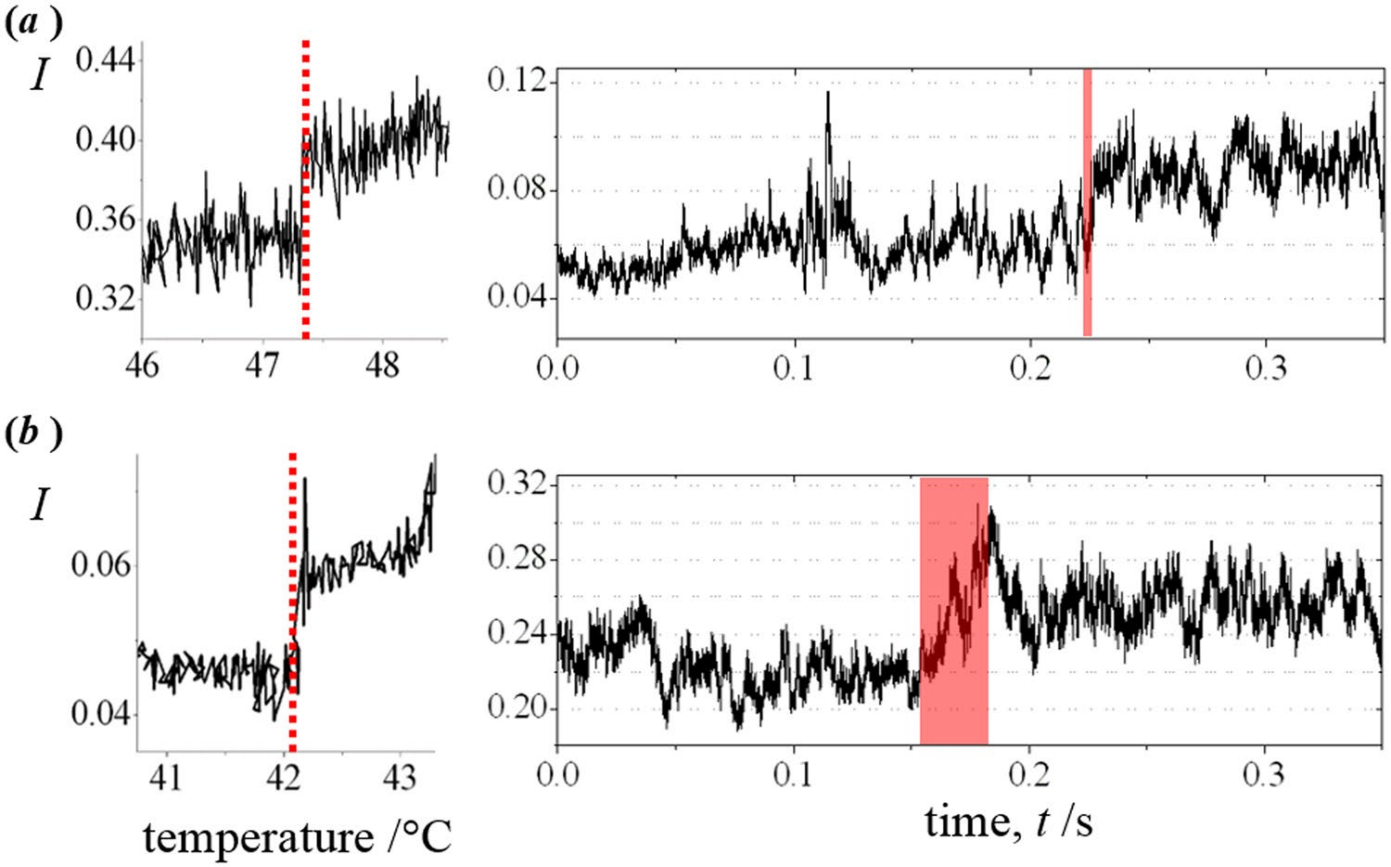
(**a**) An optically-trapped POPC/chol liposome. The full width of 4 ± 2 ms is illustrated by a red box. (**b**) An optically-trapped POPC/chol/SM liposome. Left - The back-scattered intensity of light at 1070 nm. Right - The back-scattered intensity of light at 488 nm measured, with high temporal resolution.

The gradual change in the intensity profile for the sequence of Raman spectra in 3(*a*) contrasts with the abrupt changes in the light-scattering profile as temperature is increased. Figure 4 shows the temperature dependence of the intensity of back-scattered light from an optically-trapped liposome of 1 μm-diameter for 4(*a*) POPC/chol and 4(*b*) POPC/chol/SM. Measurements were made at low temporal resolution (Left; 1070 nm), and at high temporal resolution (Right; 488 nm). Abrupt changes in the modulated intensity of scattered light were observed across 4 ± 2 and 30 ± 5 ms, respectively, for the high resolution measurements (corresponding to 0.1 °C and 1 m°C, respectively). These were observed at 42 °C and 47 °C, respectively. The change in the intensity of backscattered light from a liposome is most likely the result of deformation of the lipid bilayer in the optical-trapping laser (resembling the effect illustrated in Fig. 2); thus, a sudden change in the bending modulus of the lipid bilayer must take place on heating as a consequence of a change in the membrane fluidity (or microviscosity). The temperature values of 42 °C and 47 °C are similar to those reported for the centre of the more gradual changes in steady-state fluorescence anisotropy measurements of 1,6-diphenyl 1,3,5-hexatriene (DPH) as a function of temperature; which were used to propose phase diagrams for POPC/chol and POPC/chol/SM bilayers^25^. The light-scattering data in Fig. 4, and the fluorescence data in ref. 25 are both reporting on membrane fluidity. This could explain the consistent temperature values obtained, however, the light scattering measurement is sensitive to a more abrupt change in the bending modulus, whereas the fluorescence measurement detects a smooth change in molecular diffusion.

Label-free spectroscopic techniques, namely elastic and inelastic light-scattering, are illustrated in Figs 3 and 4. Other physical measurements of lipid bilayers can be made to monitor or infer the structural changes that take place as temperature is changed. An alternative label-free approach suitable for monitoring solid-supported lipid bilayers (SLBs) is optical birefringence^27^. The difference in the refractive indices, along perpendicular and parallel axes to the lamellar bilayer, has been shown to undergo an abrupt change across ~4 °C during the cooling of saturated phosphocholine lipid bilayers through the main transition. The change in optical birefringence is due to a decrease in bilayer thickness (*trans*-*gauche* isomerisation, ordering and tilt of lipid molecules), but comparable data has not been reported for multicomponent lipid bilayers. The fluidity of lipid bilayers can be monitored indirectly via the steady state fluorescence anisotropy of chromophores such as 1,6-diphenyl 1,3,5-hexatriene (DPH) and trans-parinaric acid. Sharp transitions, across a few °C, can be measured through the main transition, but the profiles become considerably broader with multicomponent lipid bilayers^25, 28, 29^. Similar measurements can be made by monitoring the lifetime weighted fluorescence quantum yield^25, 29^ or the relative fluorescence intensity^25, 29, 30^. Lentz *et al*. have also shown that different microviscosities can be measured for small unilamellar vesicles (SUVs) and large multilamellar vesicles in DPPC bilayers using fluorescence anisotropy measurements of DPH^31^, which was able to undergo more rapid rotations in multilamellar vesicles, compared with SUVs, below the main transition temperature. In a subsequent publication, Lentz *et al*. showed that the miscibilities of lipids in multicomponent bilayers are markedly different in SUVs compared to large multilamellar vesicles^28^. The fluorescence probe, N-[(4′-N,N-diethylamino)-3-hydroxy-6-flavonyl]methyl-N,N-dimethyloctyl ammonium bromide (F2N8), is sensitive to the degree of hydration in a lipid bilayer. M’Bate *et al*. have observed very broad changes in the fluorescence quantum yield of F2N8, across a width of >20 °C, through the gel-to-fluid transition in DPPC bilayers of large unilamellar vesicles of 100 nm-diameter. Cholesterol was also shown to decrease the degree of hydration and the temperature-dependent profiles were considerably broader^32^.

There is debate in the literature as to whether the properties of binary mixtures (such as POPC/chol) should be described by phase-separated L_o_ and L_d_ regimes, or by gradual changes in a largely homogeneous lipid bilayer^25^. Our results suggest that dynamic heterogeneities exist in POPC/Chol bilayers at room temperature consisting of transient microdomains, enriched in cholesterol, which assemble and then disassemble in a fluid bilayer; these microdomains are likely to resemble the structure of the liquid-ordered phase. The transient existence of microdomains is consistent with observations by others (for example, see^17^). By measuring the light scattering signal from an optically-trapped liposome, we have been able to probe an area of lipid bilayer corresponding to a few μm^2^ and detect an abrupt change in the bending modulus of the lipid bilayer. A strong possibility is that this abrupt change corresponds to a threshold condition for existence of dynamic microdomains in a fluid lipid bilayer. A change in the heat flow across the threshold temperature would not be expected because the microdomains assemble and disassemble reversibly at lower temperatures, however, there should be a change in the bending modulus of the bilayer when the microdomain structures can no longer exist. Hence, there was no evidence of a transition in the thermogram from differential scanning calorimetry, but an abrupt change was observed in the light scattering experiment. The timescale for the temperature-induced dissolution of ordered microdomains (2(*b*)) is approx. 10 times faster than that measured in ref. 17 for spontaneous dissolution of lipid rafts in droplet-interface bilayers.

The bending rigidity of saturated phosocholine/Chol lipid bilayers have been estimated before from measurements of undulations on giant unilamellar vesicles (GUV; 20 μm-diameter)^33^. There authors have not recorded many data points in the temperature profile shown in the publication, with a maximum recorded temperature of 40 °C (below the abrupt change in light-scattering intensity reported in Fig. 4), however, the results in [33] indicate that an abrupt change in the measured bending rigidity of a GUV is not particularly likely. The light scattering experiment, reported here, is much more sensitive to sharp transitions in the bending rigidity, even if the transition has a relatively low magnitude of change, which might be the case in POPC/Chol bilayers. As the vesicle deforms along the axis of the trapping laser, a greater area of lipid bilayer will be brought into the waist of the laser, which will result in an increase in the optical force exerted on the lipid vesicle, and hence accentuate the deformation, leading to the sharp signal response. Work by others^34^ have also shown that molecular diffusion in unsaturated phosphocholine/Chol (1:1) lipid bilayers, which should exhibit mixed Lo/Ld phase behaviour at room temperature similar to POPC/Chol, exhibits a relatively smooth change with temperature. It is quite likely that molecular diffusion is not sensitive to the dynamic assembly and disassembly of ordered microdomain structures in the lipid bilayer, whereas the bending rigidity would almost certainly be affected by the ability of the bilayer to form ordered microdomain structures.

Elastic-light scattering (see Fig. 4) and Raman spectroscopy (see Fig. 3) are sensitive to different physical changes in lipid bilayers. The Raman spectral data reports on specific changes in intermolecular forces between the lipid molecules. The lateral packing of hydrocarbon chains and rotational diffusion of lipids appear to change continuously across a broad range of temperature according to the Raman spectral profiles. The abrupt increase in elastic-light scattering intensity is attributed to a change in the shape of the liposome due to a reduction in bilayer stiffness. The magnitude of the gradient force exerted on a liposome in an optical trap is sub-pN, and this has been shown to be sufficient to deform liposomes, with a spherical diameter (ϕ) of approx. 1 μm-diameter, to bring more lipid into the trap^35^. For the binary- and ternary- mixed bilayers, the shape change has been assumed to be spherical-to-prolate (alternatively, if the liposome was already deformed in the optical trap, then there will be an increase in eccentricity of the prolate spheroid). In contrast to optical trapping experiments using 1 μm-ϕ liposomes, atomic force microscopy (AFM) of 100 nm-ϕ liposomes have shown that sub-pN forces do not result in significant deformation of the vesicle shape^36^. In this case, a 500 pN force was required to induce ~1.5% elongation of a L_β′_-DPPC liposome. The negligible deformation caused by sub-pN forces on a 100 nm-ϕ L_β′_-DPPC liposome (<0.003%) suggests that optical forces would not be sufficient to induce melting of the gel phase of a liposomal bilayer. The reduction in curvature between 100 nm-ϕ and 1 μm-ϕ liposomes results in a significant decrease in the Young’s modulus, and the sub-pN optical force is now capable of deforming the spherical shape of the vesicle. The AFM measurements in ref. 36 suggest that the Young’s modulus will change by a further factor of ~3–4 between the gel and fluid phases.

Many physical properties of the lipid bilayer, such as intermolecular interactions, volume and heat capacity (revealed by Raman spectra, dilatometry and differential scanning calorimetry, respectively) do not appear to undergo changes during the heating of lipid bilayers as sharp as that observed in the modulated intensity of elastic-scattered light. The dissolution of microdomains (lipid rafts), enriched in cholesterol and sphingomyelin, into the disordered fluid phase of the membrane can take place on millisecond timescales^17^, and this is revealed by the intensity-modulated profile of elastic back-scattered light. Changes in the structure of the lipid membrane still occur over a much longer timescale and encompass a wide range of temperature; these are revealed by the spectrally-resolved Raman scattering intensities. The methodology, described here, will reinforce future work to investigate how microdomains in lipid bilayers affect (or are affected by) membrane proteins that self assemble into large oligomeric structures, such as CDCs. 1 μm-diameter liposomes are likely to be the optimal vesicle size for the light scattering experiments, because the entire particle can be located in the waist of the 1070 nm optically-trapping laser. The methodology is likely to be less sensitive for small unilamellar vesicles due to curvature tension (and less likely to exhibit a dramatic change in the bending modulus), and would be best suited to synthetic systems rather than live cells for the same reason.

## Materials and Methods

### Preparation of lipid vesicle dispersions

The lipid reagents used were 1,2-dipalmitoyl**-**sn**-**glycero-3-phosphocholine (DPPC; P0763, Sigma Aldrich), 1-palmitoyl-2-oleoyl-sn-glycero-3-phosphocholine (POPC; 850457, Avanti Polar Lipids), cholesterol (chol; ovine wool; 700000, Avanti Polar Lipids) and sphingomyelin (SM; egg; 860061, Avanti Polar Lipids). Pure DPPC, POPC/Chol (1:1 mole ratio) and POPC/Chol/SM (1:1:1 mole ratio) lipid vesicles (liposomes) were prepared by dissolving a total mass of 10 mg of lipid in 1 ml of chloroform and dried by a stream of nitrogen gas for 2 hours. The thin lipid film was rehydrated by vortexing for 1 hour in 1 ml of phosphate-buffered saline (PBS; 160 mM NaCl, 3 mM KCl, 8 mM Na_2_HPO_4_, 1 mM KH_2_PO_4_, pH 7.4.) and the solution extruded 12 times through a 1 μm polycarbonate membrane, whilst the temperature of the suspension was maintained at 50 °C. Liposomes were subsequently stored at 4 °C and used within a week. The original suspensions were diluted 1:100 or 1:1000 in PBS. Dynamic light-scattering (Zetasizer Nano Z; Malvern, Inc.) was used to confirm the formation of monodisperse lipid vesicles with a mean diameter of approximately 1 μm (a value of the poly-dispersity index <0.2 confirmed a monodisperse suspension). Differential scanning calorimetry was performed on 20 μL samples of the liposome suspensions (see SI, Figure S10). The main transition in DPPC lipid bilayers (gel-to-fluid) was observed, but there were no features in the heat flow traces that could be attributed to phase transition for the POPC/Chol or POPC/Chol/SM bilayers.

### Optical tweezing apparatus

The experiments were performed using a custom-built inverted microscope (see SI, Figure S1). The sample stage consisted of an aluminium plate with a ring-shaped kapton-insulated flexible heater (50 mm-outer diameter, 25 mm-inner diameter; Minco Products Inc.) bonded onto the lower surface. The temperature of the aluminium plate was monitored by a K-type thermocouple. The sample stage was isolated from the body of the microscope by a 1 cm-thick delrin plate. Both the aluminium and delrin plates had a circular opening matched to the inner diameter of the heating element. An oil-immersion, infinity-corrected, objective lens (Nikon Inc., 1.25 NA, 100×) on the microscope could be brought into contact with the lower surface of a #1.5 cover glass through the circular opening. A white light LED source (400–800 nm, ThorLabs Inc.), with a condenser lens, was used to illuminate the sample. Images of the blue component of the transmitted light (400–500 nm) were recorded on a charge-coupled device (CCD; piA640–210gm, Basler Inc.).

In order to create an optical trap in the sample plane, the back aperture of the objective lens was over filled by a continuous-wave infra-red (IR) laser (1070 nm-wavelength). The lateral and axial position of the focal point of the optical trap could be adjusted by a liquid-crystal-on-silicon (LCOS) spatial-light modulator (SLM; Hamamatsu Inc.) positioned near the source of the IR beam. The LCOS-SLM was controlled using programmes encoded in LabView software (National Instruments Corp.)^37^, and enabled a trapped particle to be accurately positioned at the focal point of a second laser beam. A dichroic mirror (DM1 in Figure S1; z488rdc, Chroma Inc.) was used to combine a collimated 488 nm-wavelength laser (JDS Uniphase, FCD488; output linewidth 0.1 nm) with the IR-trapping laser; the dichroic mirror reflected >95% of the 488 nm light, and transmitted ~50% of the 1070 nm light. The beam diameter of the 488 nm-laser was matched closely to the back aperture of the objective lens. The laser powers in the sample plane were approximately 20 mW (IR) and 3 mW (visible). The power of the 488 nm laser was not sufficient for optical tweezing; and this laser was used for light scattering measurements only. Notch filters for both the 1070 and 488 nm-wavelengths were placed in front of the CCD.

A 50 μl-droplet of the dilute vesicle suspension was dispensed onto a #1.5 cover glass, located on the temperature-regulated sample stage. A single liposome from the droplet suspension was trapped by the IR-optical tweezers and a phase transition was induced by applying a temperature ramp to the sample stage from 20 to 60 °C, at approximately 0.025 °C s^−1^. The temperature ramp was achieved by maintaining an uninterrupted voltage (fixed at 25 V) and current supply to the heating element. Feedback control of the temperature ramp was avoided to minimize transient fluctuations in temperature that would result from a modulated supply voltage. The trapping laser would have a relatively minor heating effect on an isolated liposome; we predict that the trapping laser raises the temperature of the lipid bilayer by <1 °C^38^. The power of the IR laser was set to the lowest value that was capable of maintaining a trapped liposome for the duration of the temperature ramp. Due to the construction of the heated stage, the actual temperature of the trapped liposome will be lower than that measured by the thermocouple (the trapped liposome was located in a droplet above the objective lens and the thermocouple was located on the edge of the heated stage). Thus, the pre- and main transitions have been assigned to features in Fig. 1(a) which are observed at temperatures 3 to 5 °C higher than the dilatometric measurements. Further information on the accuracy of the temperature measurement, and the temperature-time profile in the experiments, is provided in the SI.

### Measurements of elastic back-scattered light

In a previous publication, the time-dependent modulation of the back-scattered light intensity from an IR laser was shown to provide information on the change in size of an optically-trapped particle and the timescale for shape relaxation of a trapped particle from a non-equilibrium state^39^. The same type of measurements were made in this work by recording the back-scattered light intensity at either 1070 nm (a 1 laser experiment) or 488 nm (a 2 laser experiment). Approximately 50%, at 1070 nm, and >95%, at 488 nm, of back-scattered light was reflected by the dichroic mirror (DM1; see SI, Figure S1). Approximately 50%, at both 1070 and 488 nm, was then reflected by a beam splitter (BS1; ThorLabs) towards a light detector. Spatial filtering of the back-scattered light removed the majority of the back-reflected light from optical surfaces; this was accomplished by focussing, using a 160 mm achromatic lens (Comar Inc.), onto an adjustable iris (ThorLabs) in the confocal image plane. The spatially-filtered light was re-collimated with a second achromatic lens of 160 mm, and then focussed by a final 160 mm achromatic tube lens onto a photodiode detector. Either the signal at 1070 nm was monitored at 0.25 s intervals by an ordinary photodiode (0.1 ms rise-time response; DET 36 A, ThorLabs), or the signal at 488 nm was monitored at 4 μs intervals by an avalanche photodiode (20 ns rise-time response; APD 130 A, ThorLabs). Recording of light scattering at the two different wavelengths enabled the phase transitions of optically-trapped liposomes to be measured with either high sensitivity, by monitoring the strong signal intensities from light scattering at 1070 nm, or high temporal resolution by monitoring the light scattering at 488 nm on a fast (visible-wavelength) photodetector. Scattering signals were sampled by a data acquisition (DAQ) module (National Instruments, NI USB-6211) at 4 samples (S) s^−1^ from the normal photodiode; and 250 kS s^−1^ from the avalanche photodiode, in which case, the intensity data was acquired by the computer in a batch of 250 kS stored in the internal buffer of the DAQ module. Whilst data corresponding to a total measurement time of 1 s was written to a text file, there was an interval of ~0.1 second when the scattering signal was not monitored.

Back-scattered light from either the 1070 nm laser, or the 488 nm laser, could be measured while the optically-trapped liposome was illuminated by the white-light LED. This means that the trapped liposome could be observed continuously by bright-field imaging for the duration of the experiment. Hence we could ensure that the light scattering measurements were made from a single-trapped particle and, if there was evidence in the images that a second liposome had entered the beam waist of the trapping laser, then the experimental data was discarded.

### Raman measurement

The combination of a pair of dichroic mirrors, DM1 and (DM2; z700dcxr, Chroma; see SI, Figure S1) enabled the Raman-scattered light, from the 488 nm laser, collected by the objective lens to be separated from the incoming laser and back-scattered light. Spatial filtering of the Raman wavelengths was accomplished by focusing, using a 160 mm achromatic lens (Comar), onto a 150 μm pinhole (Comar) in the confocal image plane. The spatially-filtered light was re-collimated with a second achromatic lens of 160 mm, and then focussed by a 50 mm achromatic lens onto the 100 μm-width entrance slits of a spectrograph with a 0.500 m imaging triple grating monochromator (Acton Research Corp., Spectra Pro 2500i; 1800 lines/mm grating; 500 nm blaze wavelength). The detector was a −80 °C cooled, back-illuminated, charge-coupled device (Princeton Instruments Inc., Pixis 100B). Spectral data points were measured in increments of 0.018 nm (~0.5 cm^−1^); the optical resolution of the spectrograph was 2 cm^−1^, and the precision of a wavenumber measurement was 0.5 cm^−1^. Accurate wavelength calibration was achieved using a Ne/Hg lamp. Acquisition time for an individual spectrum was 30 s. Lipid-Raman spectra were background subtracted to remove the signal intensity from the water-stretching bands. The microscope focus drifted during heating of the sample stage, leading to variation in signal-collection efficiency, and overall intensities could not be compared. Hence the sequences of Raman spectra were normalised in C-H stretching region between 2780 and 3030 cm^−1^.

### Multivariate analysis of Raman spectra

A multivariate-curve resolution alternate-least squares (MCR ALS) algorithm^40^ was used to deconvolve time sequences of Raman spectra into individual spectral profiles and weighted concentrations. The spectral data sets were collected continuously over >30 minutes from a single liposome, with an integration time of 30 seconds per spectrum (a total of >60 separate measurements). The chemometric treatment involved the representation of the Raman intensity profiles as columns in the matrix **D** (i.e. *n*, intensity points per spectrum, × *m*, individual spectra), which was decomposed into a weighted sum of pure spectral profiles related to the underlying components; D = CS^T^ = c_A_s_A_^T^ + c_B_s_B_^T^ + …., where **s**_***i***_ corresponds to the (normalised) intensities of the pure spectral profiles (*n* elements) and **c**_***i***_ corresponds to the concentration profiles for the sequence of recorded spectra (*m* elements). The algorithm to find the optimal fit to the experimental data employed an iterative least-squares method to minimize the error matrix, E = D − C_fit_S_fit_^T^. The calculations were performed using MATLAB software (MathWorks, Inc.). Tauler *et al*. developed the scripts and graphical-user interface^41^. An evolving factor-analysis routine provided the initial estimation of the spectra. The multivariate analysis was performed in the region of the C-H stretching band only, between 2780 and 3030 cm^−1^, where the spectra were normalised and background subtracted. A two component fit was made to the experimental spectra, with non-negative least-squares constraints on both the spectral profiles and concentrations.

## Electronic supplementary material

Supplementary Information
