# Supplementary material for: Light Scattering By Optically-Trapped Vesicles Affords Unprecedented Temporal Resolution Of Lipid-Raft Dynamics
Source: Sci Rep. 2017 Aug 17;7:8589. doi: 10.1038/s41598-017-08980-1 (PMC5561052; doi:10.1038/s41598-017-08980-1)
Supplement: Supplementary file 1 — Supplementary Information [file 41598_2017_8980_MOESM1_ESM.pdf]

Supporting Information

Light Scattering By Optically-Trapped Vesicles Affords Unprecedented Temporal Resolution Of Lipid-Raft Dynamics

Liam Collard,[a,b] David Perez-Guaita,[c] Bayan H. A. Faraj,[d] Bayden R. Wood,[c] Russell Wallis,[d,e] Peter W. Andrew[d] and Andrew J. Hudson[a*]

[a] *Department of Chemistry, University of Leicester, Leicester, LE1 7RH, United Kingdom.*

[b] *Department of Mathematics, University of Leicester, Leicester, LE1 7RH, United Kingdom.*

[c] *Department of Chemistry, Monash University, Clayton, Victoria, 3800. Australia.*

[d] *Department of Infection, Immunity and Inflammation, University of Leicester, Leicester, LE1 9HN, United Kingdom.*

[e] *Department of Molecular and Cell Biology, University of Leicester, Leicester, LE1 7RH, United Kingdom.*

I. Experimental details.

Heating rate during the light-scattering measurements

Further detail on the accuracy of the temperature measurement

II. Further examples of light-scattering profiles

1,2-dipalmitoyl-sn-glycero-3-phosphocholine, DPPC

Heating traces

Heating and cooling traces

1-palmitoyl-2-oleoyl-sn-glycero-3-phosphocholine, POPC, and cholesterol

POPC, cholesterol and sphingomyelin

III. Differential scanning calorimetric measurement of the liposome suspensions.

IV. Lipid structure-related interpretation of Raman spectra

V. Raman spectra of 1,2-dipalmitoyl-sn-glycero-3-phosphocholine, DPPC, at various temperatures.

VI. Raman spectra of an optically-trapped liposome (1:1 mixture of 1-palmitoyl-2-oleoyl-sn-glycero-3-phosphocholine, POPC, and cholesterol) as a function of temperature: The transition from a liquid-ordered phase to co-existing liquid- ordered and disordered phases.

VII. Control measurement of an optically-trapped liposome (1:1 mixture of 1-palmitoyl-2-oleoyl-sn-glycero-3-phosphocholine, POPC, and cholesterol) at fixed temperature.

I. Experimental details.

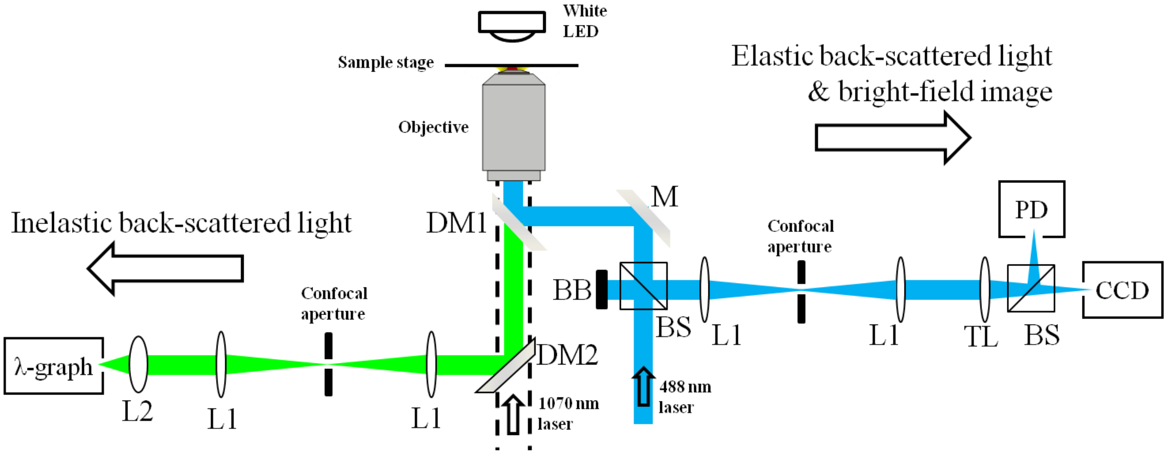

Figure S1: Schematic of the inverted-microscope apparatus. DM1, DM2 – dichroic mirrors, long-wave pass; L1 – achromatic lens, 160 mm; L2 – achromatic lens, 50 mm; TL – achromatic (tube) lens, 250 mm; M – silver mirror; BS – 50:50 beam splitter; BB – beam block; LED – light-emitting diode; CCD – charge-coupled device; PD – photodiode; λ-graph – spectrograph.

Heating rate during the light-scattering measurements

The data shown in **Figure S2** is the temperature-time data from the same experiment from which the light-scattering intensity data was used in **Figure 1(*a*)** of the original manuscript. For each measurement of the modulated light scattering intensity during the heating of an optically-trapped liposome, a new droplet of the liposome suspension was dispensed onto a cover glass. The heating rate was uncontrolled (i.e. there was feedback control from the thermocouple), however, the heating rate was still smooth, with an approximately linear rate of 0.029 °C s^{-1} in the region of the gel-to-fluid transition, which occurred at ~180s (44 °C) in **Figure 1(*a*)**, and is the region highlighted in the plot shown in **Figure S2(*b*)**.

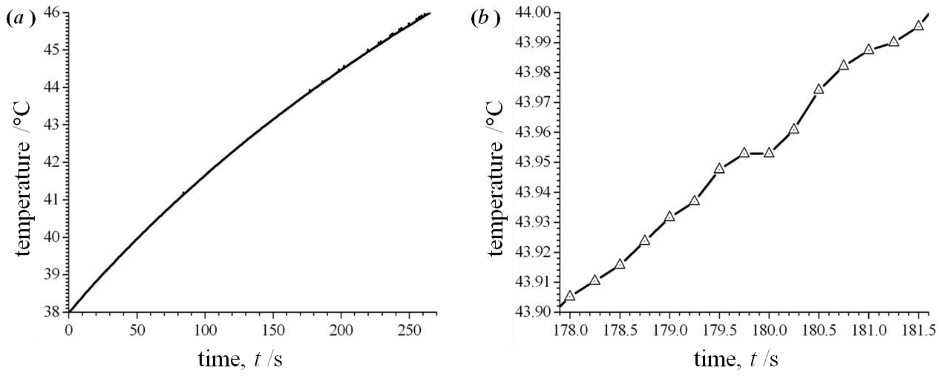

Figure S2: The profile of the temperature ramp for the experiment reported in **Figure 1(*a*)**. The gel to fluid phase transition was observed between 43.9 – 44.0 °C, approximately 180 s after the start of the experiment. The heating rate at this time was 0.029 °C s^{-1}.

Further detail on the accuracy of the temperature measurement

The design of the instrument enables the width of a phase transition to be measured on both time and temperature scales. A current limitation is that an accurate value for the actual temperature of the phase transition cannot be obtained. For example, the main transition temperature for DPPC liposomal bilayers was recorded at 44 °C (see Figure 1), wherea temperature of 41.6 °C has been determined by dilatometry.

In our experiments, the temperature is reported based on a measurement with a contact K-type thermocouple positioned at the edge of the heated aluminium plate (see **Figure S3**).

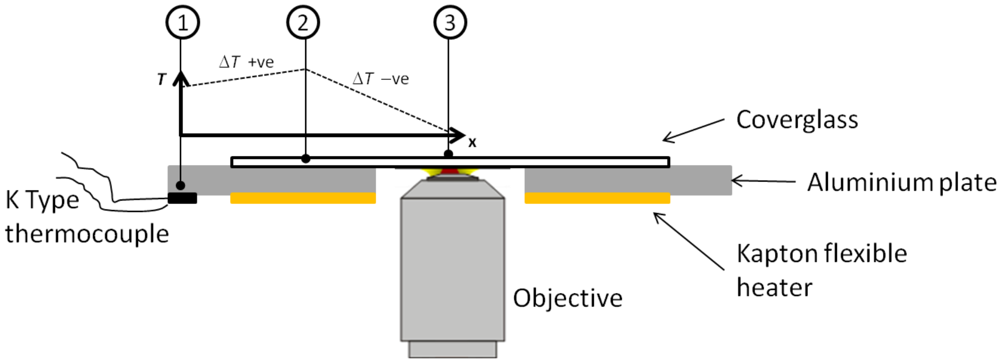

Figure S3: Schematic of the sample stage for the experimental measurements.

There was a positive temperature gradient between the edge of the aluminium plate (1) and the surface area of the aluminium plate that was actually in contact with the coverglass (2); this area of the aluminium plate is directly above the bonded-Kapton flexible element. Furthermore, there was a negative temperature gradient between the area of the coverglass in contact with the heating plate (2) and the centre of the coverglass (3); the liposome was optically-trapped at a height of ~50 μm above the centre of the coverglass.

An estimate of the magnitude of the temperature difference, $\Delta T_{(1)\rightarrow(3)}$, when the bonded thermocouple at the edge of the aluminium plate reports a temperature, T_3, of 44 °C can be made; *i.e.* the temperature at which the main phase transition of the DPPC liposomal bilayer was observed (see Figure 1).

- $\Delta T_{(1)\rightarrow(2)}$: Estimated by placing a 2nd (non-bonded) K-type thermocouple in contact with the upper surface of the aluminium plate at (2). When the bonded thermocouple reported a

temperature of 44.0 °C at (1), the non-bonded thermocouple reported a temperature of 42.2 °C at (2). Thus, $\Delta T_{(1)\rightarrow(2)}$ is ~1.8 °C, when T_1 = 44 °C.

- The coverglass can be assumed to be in good thermal contact with the aluminium plate, and the surface of the coverglass at (2) is at the same temperature as the aluminium plate.
- $\Delta T_{(2)\rightarrow(3)}$: Estimated from measurement of temperature at (2) and (3) using a non-contact IR thermometer. Absolute temperature values cannot be measured directly by the IR thermometer because the emissivity (e) of the surface of the coverglass is unknown. A linear dependence was assumed between $\Delta T_{(1)\rightarrow(2)}$ and the magnitude of T_1 above room temperature; *i.e.* $T_2 = T_1 + 1.8$ °C $\times\{(T_1 - 22)/22\}$. The emissivity of the coverglass surface was estimated by assuming the energy (E) radiated by the surface, per second per unit area, follows Stefan Boltzmann's law, $E = \sigma e(T_2)^4$, where σ is Stefan-Boltzmann's constant. $\Delta T_{(2)\rightarrow(3)}$ can now be determined from the difference in energy radiated by surface of the coverglass at (2) and (3).

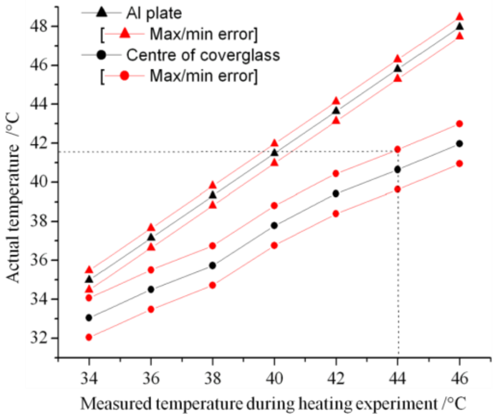

Figure S4: Estimated temperatures at (2) and (3) as a function of the reported temperature given by the bonded thermocouple at (1).

The actual temperature at (1) {Al plate} and (2) {centre of coverglass} is shown in **Figure S4** as a function of the temperature reported by the bonded thermocouple. The literature value for the main transition in DPPC bilayers (41.6 °C) is shown relative to the reported temperature of 44 °C at (1). The precision of a temperature measurement by the infrared thermometer at (2) was ± 0.5 °C. The error reported in Fig. S2 for T_3 takes into account the uncertainty in the emissivity value and the precision of T_2.

To achieve a more accurate temperature calibration in the future, we intend to make a simultaneous measurement of a temperature-sensitive spectroscopic band. For instance, the Raman band for O-H stretching in water shows a temperature dependence that could be monitored alongside the elastic-

light scattering intensity.[1] However, the modulation of the O-H stretching band with temperature is subtle. A more realistic approach would be the addition of a temperature-sensitive fluorescent probe to the aqueous phase.

II. Further examples of light-scattering profiles

(a) *1,2-dipalmitoyl-sn-glycero-3-phosphocholine, DPPC*

Heating traces

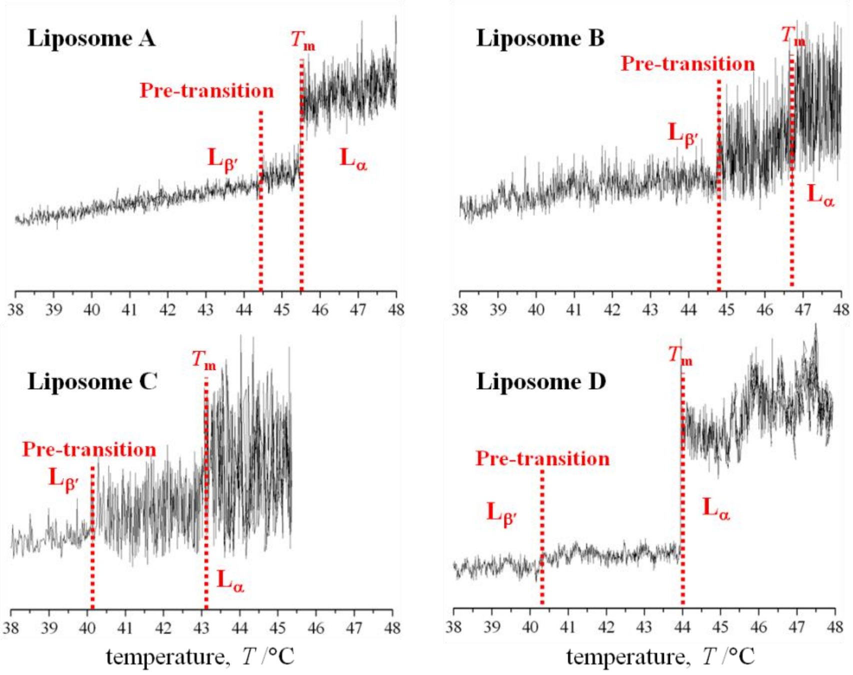

Figure S5: The profile of the back-scattered intensity, at 1070 nm, measured for 4 different optically-trapped DPPC liposomes during the heating stage of a temperature ramp. The data for Liposome D is also shown in **Figure 1(*a*)**.

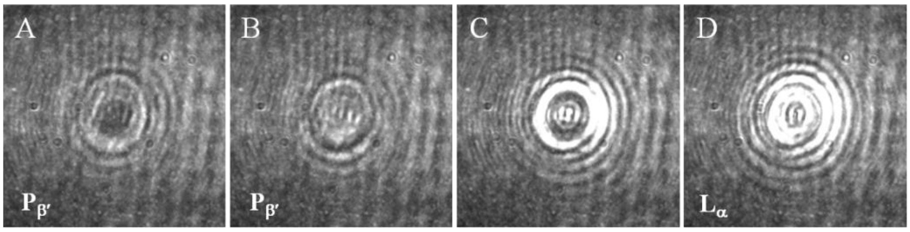

Figure S6: Images of the back-scattered light, at 1070 nm, from an optically-trapped DPPC liposome. Snapshots of the diffraction pattern of scattered light were recorded at 1 s interval during a temperature ramp. The images shown were recorded **(A)** and **(B)** immediately before, **(C)** during and **(D)** immediately after, a dramatic change in the overall scattering intensity was observed; i.e. across the $P_{\beta'} \rightarrow L_\alpha$ transition.

Heading and cooling traces

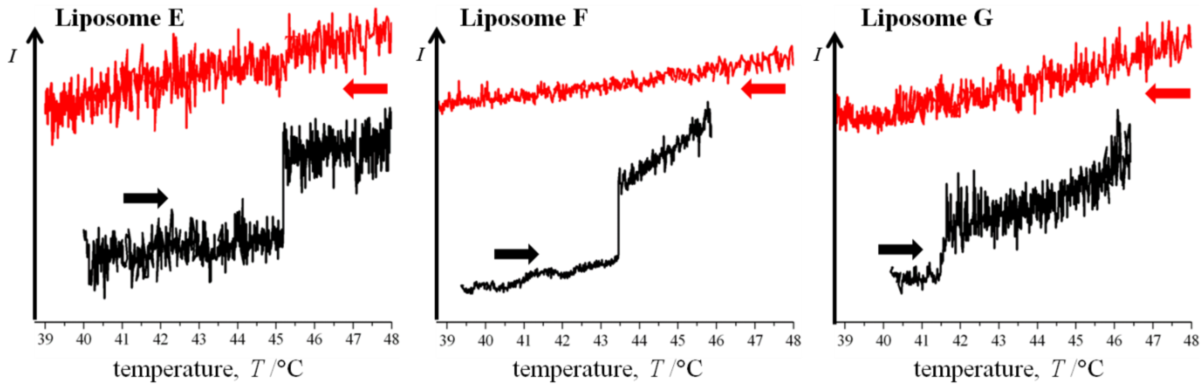

Figure S7: The profile of the back-scattered intensity, at 1070 nm, measured for 3 different optically-trapped DPPC liposomes during the heating and cooling stages of a temperature cycle.

(b) ***1-palmitoyl-2-oleoyl-sn-glycero-3-phosphocholine, POPC, and cholesterol***

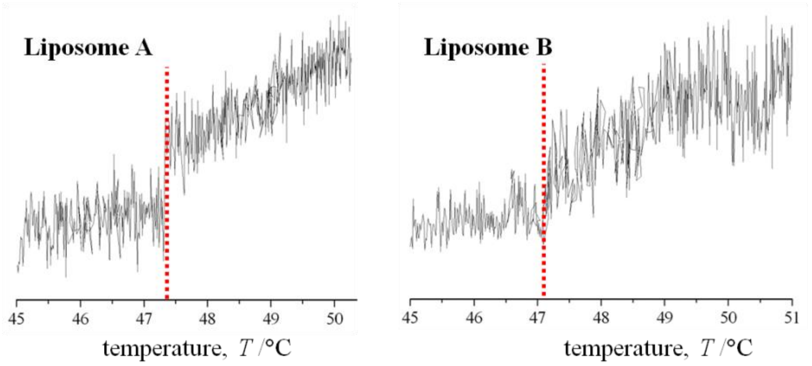

Figure S8: The profile of the back-scattered intensity, at 1070 nm, measured for 4 different optically-trapped POPC/cholesterol liposomes during the heating stage of a temperature ramp. The data for Liposome A is also shown in **Figure 4(*a*)**.

(c) ***POPC, cholesterol and sphingomyelin***

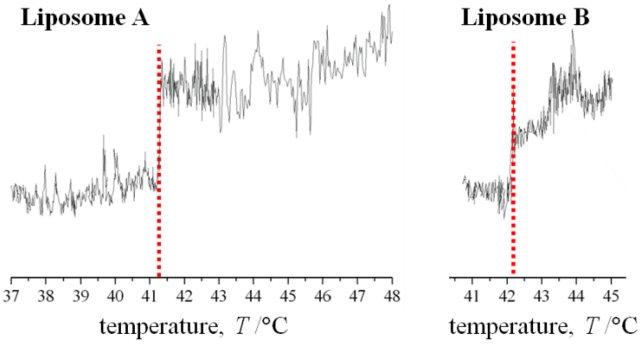

Figure S9: The profile of the back-scattered intensity, at 1070 nm, measured for 4 different optically-trapped POPC/cholesterol/sphingomyelin liposomes during the heating stage of a temperature ramp. The data for Liposome B is also shown in **Figure 4(*b*)**.

III. Differential scanning calorimetric measurement of the liposome suspensions.

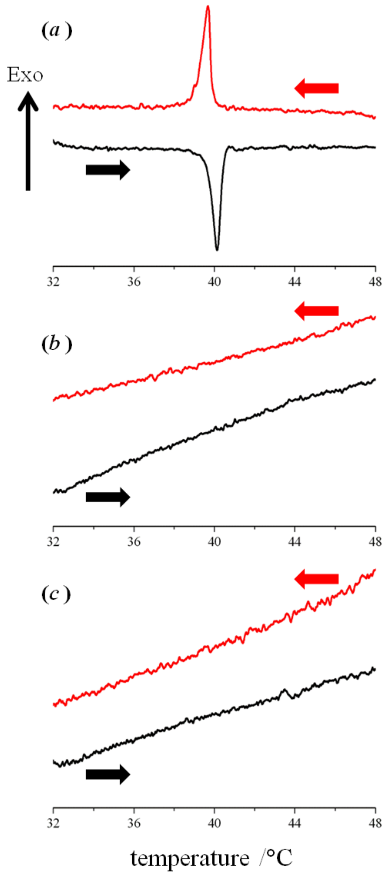

Figure S10: Differential scanning calorimetry thermograms of **(*a*)** POPC, **(*b*)** POPC/chol and **(*c*)** POPC/chol/sphingomyelin. A 20 μg sample of the concentrated suspension of liposomes was used; the scan rate was 0.5 °C/min. [DSC 1, STAR^{e} System, Mettler-Toledo Ltd.]

IV. Lipid structure-related interpretation of Raman spectra

Our work has focussed on the C-H band, which is by far the strongest feature in lipid-Raman spectra. However, we will start by giving some background on other spectral regions. The main features in Raman spectra of lipid molecules are analogous to those of long chain *n*-alkanes. There are distinct Raman bands within the C-C stretching region corresponding to tight and loose packing of the hydrocarbon chains in different phases, namely: the asymmetric and symmetric C-C stretching bands at c.1060 and 1130 cm^{-1}, which originate from all-trans C-C bonds; and the skeletal C-C stretching band at c.1080 cm^{-1} from gauche segments of the alkyl chain. The relative intensity of the bands at c.1130 and 1080 cm^{-1} has been used to estimate the number of trans-conformational segments per alkyl chain, which would be higher in the L_o phase relative to the L_d phase.[2] The intensities of all the

bands in the C-C stretching region are markedly lower than the bands in the C-H region, which is the reason why the latter was recorded in our experiments.

In a further spectral region, a blue-shift in the CH_2 twisting mode at c.1300 cm^{-1} is also consistent with the disordered phase of lipid bilayers and structures that contain a broader distribution of gauche rotamers. The packing of the hydrocarbon chains also strongly affects the relative intensity of bands for the CH_2 twisting mode and the CH_2 scissor mode at 1440 cm^{-1} but, unlike bands in the C-C stretching region, these methylene bands are not unique for trans and gauche segments, i.e. ordered and disordered lipid structures. The frequencies of the bending and stretching methylene modes are sensitive to the number of gauche segments. This is due to increasing steric repulsion between neighboring chains. However, the blue shift can be difficult to resolve in the C-H stretching region due to the overlapping band structure. All the methlyene bands in this region are much weaker than the C-H stretching bands. The CH_2 wagging mode at c.1370 cm^{-1} is an extremely weak band.

Our work has focussed on the C-H band, which is by far the strongest feature in the lipid-Raman spectrum. There are a similar number of peaks in the C-H band for different lipid and hydrocarbon molecules.[3] The C-H region comprises the symmetric (d^+; *c*. 2840 cm^{-1}) and antisymmetric (d^-; *c*. 2870 cm^{-1}) methylene stretch, the Fermi resonance of the symmetric methyl stretch (r_{FR}^+; *c*. 2920 cm^{-1}) and the antisymmetric methyl stretch (r^-; *c*. 2960 cm^{-1}).[4] A weak band is also observed at 3030 cm^{-1} for the antisymmetric CH_3 stretch of the choline head group. The intensity ratio between the d^- and d^+ bands, or, alternatively, the d^- and r_{FR}^+ bands, has been reported in the literature to provide a qualitative measure of short-range packing order of the hydrocarbon chains, where a larger ratio is indicative of greater order (alternatively the ratio has been used to measure intermolecular interactions or vibrational/torsional motion of the lipid chain).[5,6] It should be noted that the integrated intensity of the d^- and d^+ band are not believed to be affected by the relative number of trans and gauche segments, and only the relative intensity of the peak maxima. With a larger number of gauche segments, the d^+ band is broadened with substantial intensity gained at c.1855cm^{-1}.[5] The d^- frequency has also previously been associated with the extent of chain decoupling and rotational diffusion of lipids. As the number of gauche segments increases, the d^- band broadens in the spectrum and merges with the Fermi resonance band. Thus, the quantitative significance of the d^-/d^+ ratio, which some researchers refer to as the order parameter, is uncertain.

Although the d^+, d^-, r_{FR}^+ and r^- peaks are observed in the C-H band for different lipid molecules, the intensity ratios are different, which further impacts on any ability to deduce the degree of trans-gauche isomerisation in different bilayer structures. The individual spectra for phosphocholine, cholesterol and sphingomyelin molecules can be found in the literature, for example see Ref..7. Thus, the change in the membrane order, via the d^+/d^- ratio, can only be considered for a bilayer of fixed composition. Although the focus in the C-H stretching region for Raman studies of lipid bilayers has often is on 4 peak maxima in the C-H band, other researchers have shown that the entire band can be

fitted with a larger number of underlying components; for example, see Ref. 8 where the C-H was fitted with 8 underlying Lorentzian profiles. We consider that the most successful deconvolution of the C-H band has been achieved by multivariate methods; see Refs. 9 and 10.

V. Raman spectra of 1,2-dipalmitoyl-sn-glycero-3-phosphocholine, DPPC, at various temperatures.

Figure S11 shows the spectrum of inelastic (Raman) scattered-light from an optically-trapped DPPC liposome measured in the gel ($L_{\beta'}$), ripple ($P_{\beta'}$) and fluid (L_{α}) phases. The C-H region comprises the symmetric (d^+) and antisymmetric (d^-) methylene stretch, the Fermi resonance of the symmetric methyl stretch (r_{FR}^+) and the antisymmetric methyl stretch (r^-). The assignment and notation is in accord with Ref. (7). A weak band is also observed at 3030 cm^{-1} for the antisymmetric CH_3 stretch of the choline head group. The intensity ratio between the d^- and d^+ bands, or, alternatively, the d^- and r_{FR}^+ bands, has been reported in the literature to provide a qualitative measure of short-range packing order of the hydrocarbon chains, where a larger ratio is indicative of greater order.[9] The decrease in both of these ratios above the main transition for a single DPPC liposome is consistent with previous measurements on planar supported lipid bilayers[11], heterogeneous dispersions of lipids,[8] multilamellar vesicles,[12] and optically-trapped unilamellar vesicles.[13]

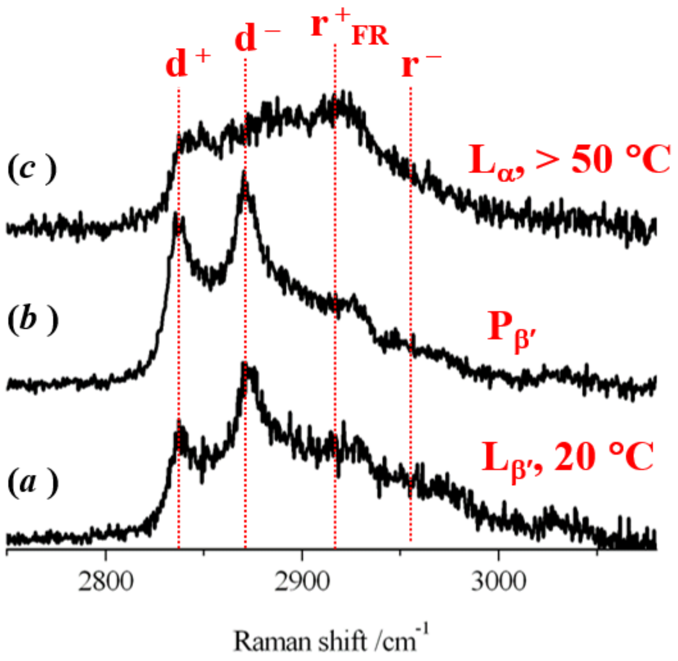

Figure S11: The Raman spectrum of an optically-trapped DPPC liposome in the region of the C-H stretching band, ***(a)*** at approx. 20 °C (the gel phase, $L_{\beta'}$), ***(b)*** at intermediate temperature (the ripple

phase, $P_{\beta'}$), and ***(c)*** at approx. 50 °C (the fluid phase, L_α). Spectra were recorded from different liposomes.

VI. Raman spectra of an optically-trapped liposome (1:1 mixture of 1-palmitoyl-2-oleoyl-sn-glycero-3-phosphocholine, POPC, and cholesterol) as a function of temperature: The transition from a liquid-ordered phase to co-existing liquid- ordered and disordered phases.

A binary mixture of a phosphocholine lipid and cholesterol can exist in a liquid-ordered phase (L_o) sharing characteristics of gel ($L_{\beta'}$) and fluid (liquid-disordered, L_d) phases. While the intercalation of cholesterol with phosphocholine molecules disrupts the planar triangular lattice in the gel phase, a *trans* conformation of the hydrocarbon chains is more favoured in the fluid phase resulting in a higher degree of short-range order. A 1:1 mole ratio of lipid components, at 20 °C, will produce a bilayer in the L_o phase. The L_o and L_d phases are expected to co-exist in bilayers at either lower mole fractions of cholesterol or, alternatively, at higher temperature.[13]

In **Figure S12(*a*)**, the Raman spectrum of an optically-trapped POPC/cholesterol liposome is shown as a function of temperature. All the spectra were recorded from the same trapped vesicle at regular intervals of time during a temperature ramp from 20 to 60 °C (80 spectra, 30 s intervals; approx.-linear ramp of +0.025 °C s^{-1}). The d^+, d^-, r_{FR}^+ and r^- bands can be distinguished in the broad C-H region, but the spatial resolution of the individual bands is not as clear as that for a pure DPPC liposome. The change in the structure of the lipid bilayer between room temperature, where a pure L_o phase should exist, and high temperature, where L_o and L_d phases should co-exist, appears to result in a small increase in the ratio d^- / d^+. This is not the direction of change that was expected for the ratio of the CH_2 stretching bands. An increase in the ratio is usually observed with greater short-range order of the hydrocarbon chain packing;[5] thus, a decrease in the ratio d^- / d^+ was expected following the $L_o \rightarrow L_o/L_d$ transition. Although there is considerable analysis and interpretation of the temperature-dependence of Raman spectra in the vicinity of the main transition in lipid bilayers, there is relatively sparse reported data on the fluid phases of multicomponent systems. Surovtsev et al. have recorded Raman spectra between 50 and 315 K for bilayers comprising a 1:1 mole ratio of POPC and cholesterol.[14] They observed a gradual decrease in the ratio, d^-/d^+, between 200 and 315 K (from 1.3 to just below 1.0). We have observed, in Figure S4 ***(a)***, an increase in d^-/d^+ from approximately 0.95 to 1.10, between 293 and 333 K. The significance of the increase, apparent in **Figure S12(*a*)**, indicates that it is not strictly reliable to associate the relative intensities of Raman peaks with the physical properties of lipid membranes.

Although the change in ratio d^- / d^+ with temperature for a POPC/cholesterol bilayer does not conform to changes observed for other bilayer compositions, a shift in the d^- band to higher frequency can be

seen in the raw spectral profiles in **Figure S12(*a*)** which is consistent with interpretation in the older literature. The d^- frequency has previously been associated with the extent of chain decoupling and rotational diffusion of lipids in bilayers.[5] Furthermore, there is a substantial increase in the intensity of the r_{FR}^+ band relative to the d^- band in **Figure S12(*a*)**, which has also been used as an indicator of packing disorder.[5]

The results of multivariate curve resolution on the sequence of Raman spectra from **Figure S12(*a*)** is shown in **S12(*b*)** and **(*c*)**. A fitting of two components captured 99.0% of the variance in the experimental data. In **S12(*d*)**, the residual is shown for a representative example from the sequence of experimental spectra shown in **S12(*a*)**. The residual in **S12(*d*)** corresponds to 19 minutes, where the component concentrations are approximately equal. The signal remaining in **S12(*d*)** appears to be stochastic, without any trace of the C-H stretching band, and hence supports the suitability of the multivariate model. The chemometric analysis suggests that the Raman intensity profile changes continuously, and smoothly, between 20 and 60 °C. The fitted components, **A** and **B**, bear a close resemblance to the initial and final experimental spectra, respectively. The gradual change in the intensity profile for the sequence of Raman spectra in **Figure S12(*a*)** contrasts with the discrete change in the light-scattering profile assigned to the $L_o \rightarrow L_o/L_d$ transition temperature in Figure 2(*a*). Hence elastic-light scattering and Raman spectroscopy are sensitive to different physical changes in lipid bilayers. While a discrete phase transition can be determined by elastic-light scattering, the changes in ensemble-averaged properties of the lipid molecules in the bilayer are reported in the Raman spectra. The ensemble averages for the lateral packing of hydrocarbon chains and rotational diffusion of lipids appear to change continuously across a broad range of temperature near the phase boundary according to the Raman spectral profiles.

A gradual change in the Raman spectra was also reported by Surovtsev et al. across a broad range of temperature encompassing the gel-to-fluid transition for lipid bilayers containing cholesterol.[19] Although it is known that the temperature of the gel-to-fluid transition is lowered and broadened in the presence of cholesterol,[15] the full range of temperatures (from 200 to 315 K) over which the Raman intensities were seen to change in Ref. 19 is still much wider than the expected broadening of the gel-to-fluid transition.

There is debate in the literature as to whether the properties of binary mixtures of lipids should be described by phase-separated L_o and L_d regimes, or by gradual changes in a largely homogeneous lipid bilayer.[20] By measuring the light scattering signal from an optically-trapped liposome, we have been able to probe an area of lipid bilayer corresponding to a few μm^2 and detect heterogeneities due to differences in light scattering from the L_o and L_d phases, which have been difficult to characterise using conventional thermal analysis techniques or Raman spectroscopy.

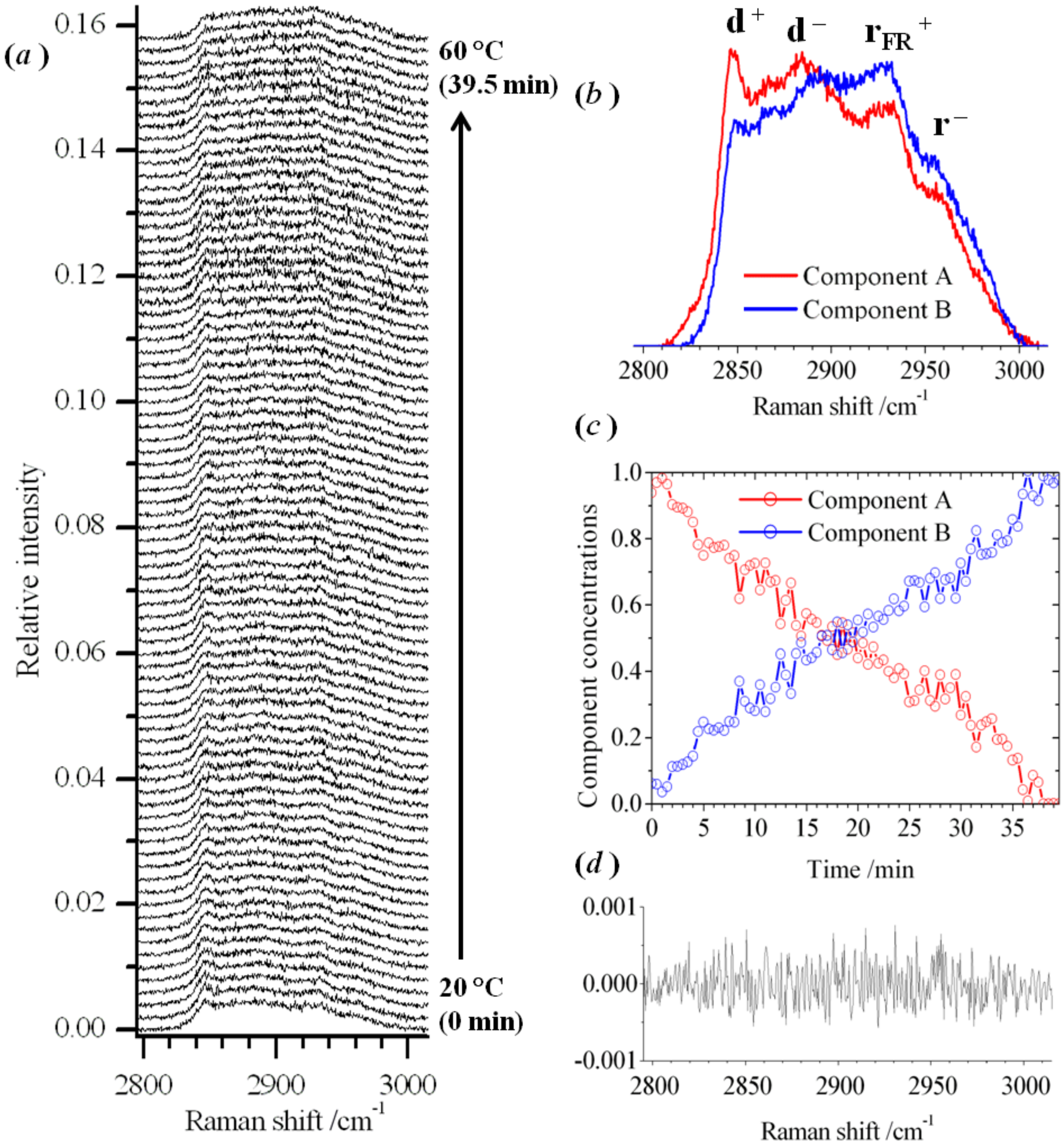

Figure S12: Raman spectra of an optically-trapped POPC/Chol liposome, in the region of the C-H stretching band. **(*a*)** A sequence of experimental Raman spectra recorded at 30 s intervals. The overall duration of the experiment was 39.5 minutes. An unregulated-temperature ramp, between 20 and 60 °C, was applied to the microscope stage. **(*b*)** and **(*c*)** The pure spectral profiles and concentration profiles for two components obtained by multivariate curve resolution. **(*d*)** The residual for the 37th spectrum, recorded after 19 minutes, following optimisation of the component profiles.

VII. Control measurement of an optically-trapped liposome (1:1 mixture of 1-palmitoyl-2-oleoyl-sn-glycero-3-phosphocholine, POPC, and cholesterol) at fixed temperature.

A sequence of Raman spectra was recorded from an optically-trapped POPC/cholesterol vesicle maintained at ambient temperature. The raw spectral data are shown in **Figure S13(*a*)**. The spectra were recorded from the same trapped vesicle at regular intervals of time (36 spectra, 30 s intervals).

No change in the frequencies or intensities of peaks in the C-H stretching region was observed at constant temperature. The results of multivariate curve resolution on the sequence of Raman spectra from **Figure S13(*a*)** is shown in **(*b*)** and **(*c*)**. The spectral data set was fitted with two components, using the same approach made to analyse the temperature-dependent Raman spectra (see **Figures 3** and **S12**). A fitting of two components captured 99.7% of the variance in the experimental data, however, the fitted components, **A** and **B**, show nearly no change in the relative intensities and positions of the d^+, d^-, r_{FR}^+ and r^- bands. Due to a change in the overall intensity of the recorded Raman spectra, which is the result of focus drift during the course of the experiment, the components **A** and **B** reflect the small difference in the recorded spectra following background subtraction, and the component weighting still shows a gradual change between **A** and **B** as a function of time. In **Figure S13(*d*)**, the residual is shown for a representative example from the sequence of experimental spectra in **S13(*a*)**.

Raw spectral data are superimposed in **Figure S14** illustrating the initial and final measurements for the control experiment (fixed temperature in **(*a*)**) and the heating experiment (in **(*b*)**; reproduced from Figure 3).

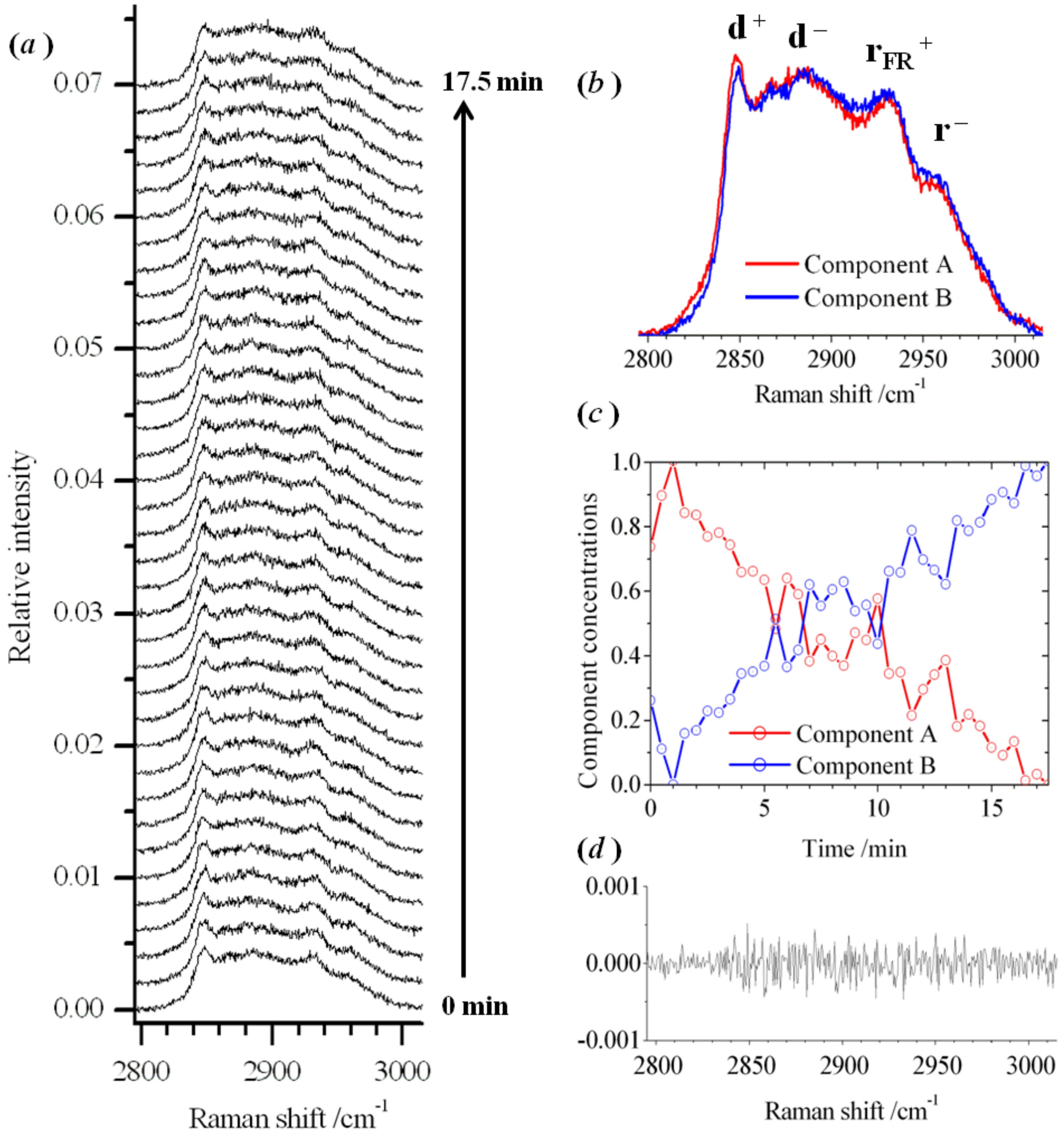

Figure S13: Raman spectra of an optically-trapped POPC/Chol liposome (1:1 mol ratio), in the region of the C-H stretching band. ***(a)*** A sequence of experimental Raman spectra recorded at 30 s intervals. The overall duration of the experiment was 17.5 minutes. The microscope stage was maintained at ambient temperature. ***(b)*** and ***(c)*** The pure spectral profiles and concentration profiles for two components obtained by multivariate curve resolution. ***(d)*** The residual for the 17th spectrum, recorded after 8 minutes, following optimisation of the component profiles.

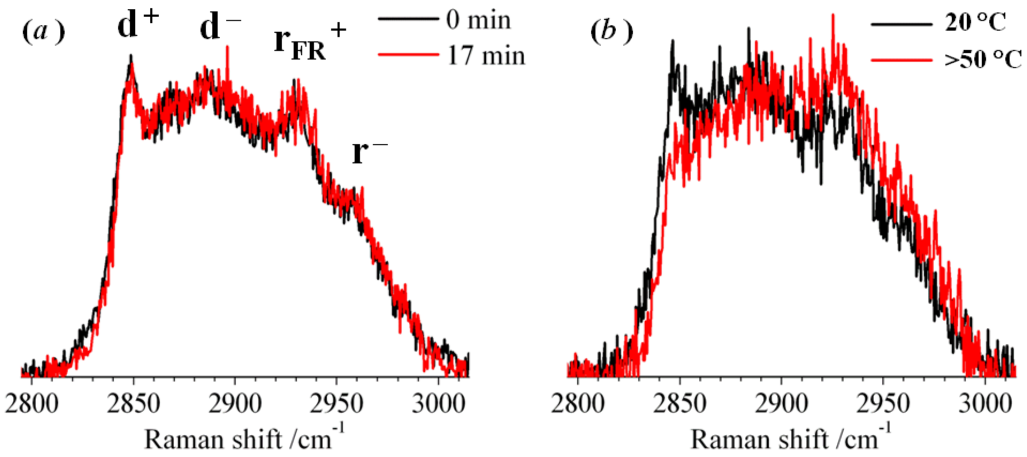

Figure S14: Raman spectra of an optically-trapped POPC/Chol liposome (1:1 mol ratio), in the region of the C-H stretching band. **(*a*)** The vesicle was maintained at ambient temperature; the spectra shown correspond to the first and last measurement in Figure S1. **(*b*)** The temperature was increased from ambient conditions to >50 °C; the spectra shown correspond to the first and last measurement in the raw spectral data in Figure 3.

[1] Artlett, C. P. & Pask, H. M. Optical remote sensing of water temperature using Raman spectroscopy. *Optics Express* **23**, 31844-31856 (2015).

[2] Wong, P. T. T. Raman Spectroscopy of Thermotropic and High-Pressure Phases of Aqueous Phospholipid Dispersions. *Ann. Rev. Biophys. Bioeng.* **13**, 1-24 (1984).

[3] Czamara, K., Majzner, K., Pacia, M. Z., Kochan, K., Kaczor, A. & Baranska, M. Raman spectroscopy of lipids: a review. *J. Raman Spectroscopy* **46**, 4–20 (2015).

[4] MacPhail, R. A., Strauss, H. L., Snyder, R. G. & Ellinger, C. A. C-H Stretching Modes and the Structure of n-Alkyl Chains. 2. Long, All-Trans Chains. *J. Phys. Chem.* **88**, 334-331 (1984).

[5] Gaber, B. P., Yager, P. & Peticolas, W. L. Interpretation of biomembrane structure by Raman difference spectroscopy. *Biophys J.* **21**, 161-176 (1978).

[6] Larsson, K. R. & Rand, R. P. Detection of changes in the environment of hydrocarbon chains by Raman spectroscopy and its application to lipid-protein systems. *Biochim. Biophys. Acta.* **326**, 245-255 (1973).

[7] Ando, J., Kinoshita, M., Cui, J., Yamakoshi, H., Dodo, K., Fujita, K., Murata, M. & Sodeoka, M. Sphingomyelin distribution in lipid rafts of artificial monolayer membranes visualized by Raman microscopy. *Proc. Natl. Acad. Sci. U S A* **112**, 4558–4563 (2015).

[8] Potma, E. O. & Xie, X. S. Detection of single lipid bilayers with coherent anti-Stokes Raman scattering (CARS) microscopy. *J. Raman Spectroscopy* **34**, 642–650 (2003).

[9] Schaefer, J. J., Ma, C. & Harris, J. M. Confocal Raman microscopy probing of temperature-controlled release from individual, optically-trapped phospholipid vesicles. *Anal. Chem.* **84**, 9505-9512 (2012).

[10] Fox, C. B., Uibel, R. H. & Harris, J. M. Detecting Phase Transitions in Phosphatidylcholine Vesicles by Raman Microscopy and Self-Modeling Curve Resolution. *J. Phys. Chem. B* **111**, 11428-11436 (2007).

[11] Lee, C. & Bain, C. D. Raman spectra of planar supported lipid bilayers. *Biochim. Biophys. Acta.* **1711**, 59-71 (2005).

[12] Dmitriev, A. A. & Surovtsev, N. V. Temperature-Dependent Hydrocarbon Chain Disorder in Phosphatidylcholine Bilayers Studied by Raman Spectroscopy. *J. Phys. Chem. B* **119**, 15613-15622 (2015).

[13] de Almeida, R. F. M., Fedorov, A. & Prieto, M. Sphingomyelin/Phosphatidylcholine/Cholesterol Phase Diagram: Boundaries and Composition of Lipid Rafts. *Biophys. J.* **85**, 2406-2416 (2003).

[14] Surovtsev, N. V. & Dzuba, S. A. Flexibility of phospholipids with saturated and unsaturated chains studied by Raman scattering: The effect of cholesterol on dynamical and phase transitions. *J. Chem. Phys.* **140**, 235103 (2014).

[15] Halling, K. K. & Slotte, J. P. Membrane properties of plant sterols in phospholipid bilayers as determined by differential scanning calorimetry, resonance energy transfer and detergent-induced solubilisation. *Biochim. Biophys. Acta.* **1664**, 161–171 (2004).
